# Surgery for hip preservation—let the patient decide

**DOI:** 10.1093/jhps/hnw041

**Published:** 2016-11-10

**Authors:** Richard Villar

**Affiliations:** Editor-in-Chief, JHPS

I got to thinking the other day. Thinking what might have happened had hip arthroscopy never existed. Around the world I see colleagues being threatened by Governments, insurers, even departmental heads who should know better. The basic tenet of the threats is that impingement is unproven surgery and, as a consequence, should not be undertaken until proof exists. So, my first question is simple—what do we (or they) mean by proof? Readers of *JHPS* may have read a recent Editorial,^1^ which addressed this very issue. There appears to be a view that only the procedure with the most successful result is the one that should be offered. Yet how valid a position is this? It must surely depend on the patient; patients, as many might agree, are at best unpredictable.

For example, if one operation carries a 95% chance of success and another only 65%, does that mean the 65% procedure is contraindicated? Let us look more closely. First, our definition of success, and our derivation of 95%, may not be the same as a patient’s and second, by what right do we discount a lower score? Take osteoarthritis, or for that matter dysplasia, as examples. The party line is that hip arthroscopy should not be undertaken in the presence of osteoarthritis nor, for that matter, in dysplasia. I have seen blood almost spilt at meetings when this matter is debated. And yet there are numerous papers now published that declare symptoms can be improved in both these conditions by the use of hip arthroscopy.

Looking closer still, we know that an arthroplasty undertaken for osteoarthritis of the hip has a probable 95% chance of improving a patient’s symptoms. We also know that a hip arthroscopy has, roughly, a 55% chance of doing the same, albeit for a limited time period. Does that mean hip arthroscopy should be excluded as a considered option? I think not but admit that is a purely personal view. I believe it is our responsibility as surgeons to lay out our stall and to discuss with our patients what is a suitable solution. One patient may select the 55% option, another the 95%. Ultimately it is a surgeon-advised, patient-decided conclusion that is reached. The patients are our masters and as few obstructions as possible should lie between patient and surgeon. Any non-medic who stands between these two key individuals should, in my view, hang their head in shame, assuming ethical practice, of course.

So, I worry when I hear colleagues from around the world being asked to attend meetings with healthcare administrators, at times politicians, sometimes even each other, in order to justify the role of hip preservation surgery, arthroscopy in particular. As an observer it appears there is only one issue at stake, a desire by purse-string holders to save money. Surgeons have perhaps not helped themselves through the huge, 400% increase in the diagnosis and management of femoroacetabular impingement in the last ten years,^2^ a fact that led to the development of the so-called Warwick Agreement,^3^ remarkable not so much for its conclusions but that it brought together so many different interested parties from around the world. The challenge to hip preservation affects us all, but the patient in particular. It is our task as practitioners to protect our patients and to keep those whose sole mission is to save money from muddying the waters. I find it odd that the matter needs to be discussed at all when the UK’s National Institute for Health and Care Excellence (NICE) concluded 5 years ago that “Current evidence on the efficacy of arthroscopic femoroacetabular surgery for hip impingement syndrome is adequate in terms of symptom relief in the short and medium term.”^4^ Has so much changed? I doubt it. Other than the desire to save money, of course. To me the solution is very simple; let the patient decide.

Turning to the last issue (number 3.3) of *JHPS*, this was again filled with more information than any editorial can reasonably summarize. The review by Bech *et al*.,^5^ I admit, went straight to our anaesthetic team, as reassurance that they are not alone in their management of pain after hip arthroscopic surgery. Meanwhile the paper by Hujazi *et al*.^6^ on the normal ischiofemoral distance definitely caught many eyes, as ischiofemoral impingement becomes more widely diagnosed in our various clinical practices.

And as for this issue, number 3.4, where does an Editor-in-Chief begin? I am spoilt for choice. That said, I did especially like the paper by Kivlan *et al.*^7^ on defining the greater trochanter-ischial space. Impingement in that area was not a problem I had actually considered and kick myself for not doing so. Thanks to these three very capable authors for bringing the matter to our attention. And while discussing hitherto undescribed phenomena, how about the paper by Schröder *et al*.^8^ on their so-called “hip vacuum sign”, a new radiographic finding in FAI? I am straight back to all those frog-leg lateral radiographs to see what I might have missed.

As with earlier issues, *JHPS* is once again filled to the hilt with pearls and I commend each and every one to you.

My very best wishes to you all.
